# CCCTC-binding factor inhibits breast cancer cell proliferation and metastasis via inactivation of the nuclear factor-kappaB pathway

**DOI:** 10.18632/oncotarget.18977

**Published:** 2017-07-04

**Authors:** Jie Wu, Peng-Chang Li, Jun-Yi Pang, Guo-You Liu, Xue-Min Xie, Jia-Yao Li, Yi-Cong Yin, Jian-Hua Han, Xiu-Zhi Guo, Ling Qiu

**Affiliations:** ^1^ Department of Clinical Laboratory, Peking Union Medical College Hospital, Peking Union Medical College and Chinese Academy of Medical Science, Beijing, 100730, China; ^2^ Department of Pathology, Peking Union Medical College Hospital, Peking Union Medical College and Chinese Academy of Medical Science, Beijing, 100730, China; ^3^ State Key Laboratory of Medical Molecular Biology, Department of Biochemistry and Molecular Biology, Institute of Basic Medical Sciences, Chinese Academy of Medical Sciences and Peking Union Medical College, Beijing, 100005, China

**Keywords:** CCCTC-binding factor, breast cancer, proliferation, metastasis, nuclear factor-kappaB

## Abstract

CCCTC-binding factor (CTCF) is an important epigenetic regulator implicated in multiple cellular processes, including growth, proliferation, differentiation, and apoptosis. Although CTCF deletion or mutation has been associated with human breast cancer, the role of CTCF in breast cancer is questionable. We investigated the biological functions of CTCF in breast cancer and the underlying mechanism. The results showed that CTCF expression in human breast cancer cells and tissues was significantly lower than that in normal breast cells and tissues. In addition, CTCF expression correlated significantly with cancer stage (*P =* 0.043) and pathological differentiation (*P* = 0.029). Furthermore, CTCF overexpression resulted in the inhibition of proliferation, migration, and invasion, while CTCF knockdown induced these processes in breast cancer cells. Transcriptome analysis and further experimental confirmation in MDA-MD-231 cells revealed that forced overexpression of CTCF might attenuate the DNA-binding ability of nuclear factor-kappaB (NF-κB) p65 subunit and inhibit activation of NF-κB and its target pro-oncogenes (tumor necrosis factor alpha-induced protein 3 [TNFAIP3]) and genes for growth-related proteins (early growth response protein 1 [EGR1] and growth arrest and DNA-damage-inducible alpha [GADD45a]). The present study provides a new insight into the tumor suppressor roles of CTCF in breast cancer development and suggests that the CTCF/NF-κB pathway is a potential target for breast cancer therapy.

## INTRODUCTION

Breast cancer continues to be the most common malignant tumor in women worldwide, with an estimated 25% of new cancer cases diagnosed [1–4]. Understanding the gene abnormalities that participate in the occurrence and development of breast cancer is the key to its prevention and treatment.

Nuclear factor-kappa B (NF-kB), the key transcription factor that mediates the inflammatory response, is involved in carcinogenesis-associated cellular processes, such as inflammation, proliferation, invasion, metastasis, angiogenesis, differentiation, and survival [5, 6]. Studies have shown that NF-kB regulates the expression of several responsive genes that are associated closely with tumorigenesis, such as those encoding cytokines, transcription factors, growth factors, and apoptosis regulators [7]. Aberrant activation of NF-κB occurs in most cancers, including breast cancer [8–10]. Many negative regulators, including microRNAs, long non-coding RNAs, and regulatory proteins, function as tumor suppressors by inhibiting the uncontrolled activation of NF-kB [5, 6, 11].

CCCTC-binding factor (CTCF) is an evolutionarily conserved and ubiquitously expressed zinc finger protein. CTCF was originally discovered as a candidate tumor suppressor, given its ability to bind to the promoter of the oncogene c-myc to inhibit its expression [12]. As an epigenetic regulator, CTCF regulates the expression of several genes, including tumor suppressor genes *p53* and *p16* [13, 14] and pro-apoptotic gene *bax* [15]. Studies have identified approximately 20,000 CTCF-binding sites (CBS) in the human genome [16, 17]. *CTCF* was considered as a possible target for deletions of chromosomal regions of 16q22.1 that occur commonly in breast and prostate cancers [18]. In recent years, *CTCF* has been found to be mutated sporadically in diverse cancer types, such as leukemia, breast, uterine, and prostate cancers [19–23] and is involved in inhibiting cancer cell growth and clonogenicity [24]. *CTCF* deletion has been reported in lobular carcinoma of the breast [25]. However, the exact role of CTCF in breast cancer is unclear.

In this study, we examined CTCF expression in breast cancer tissues and cell lines, and analyzed the correlation between CTCF expression and various clinicopathological parameters. Furthermore, the effects of CTCF on cell proliferation and migration were investigated through the overexpression or knockdown of *CTCF* in breast cancer cells. Transcriptome microarray analysis and validation experiments suggested that CTCF might inhibit the activation of the NF-κB (p65) subunit and its target genes. Our experiments imply an important role for CTCF in inhibition of NF-kB over-activation in breast cancer occurrence and metastasis.

## RESULTS

### CTCF is downregulated in breast cancer tissues and cell lines

**Figure 1:**
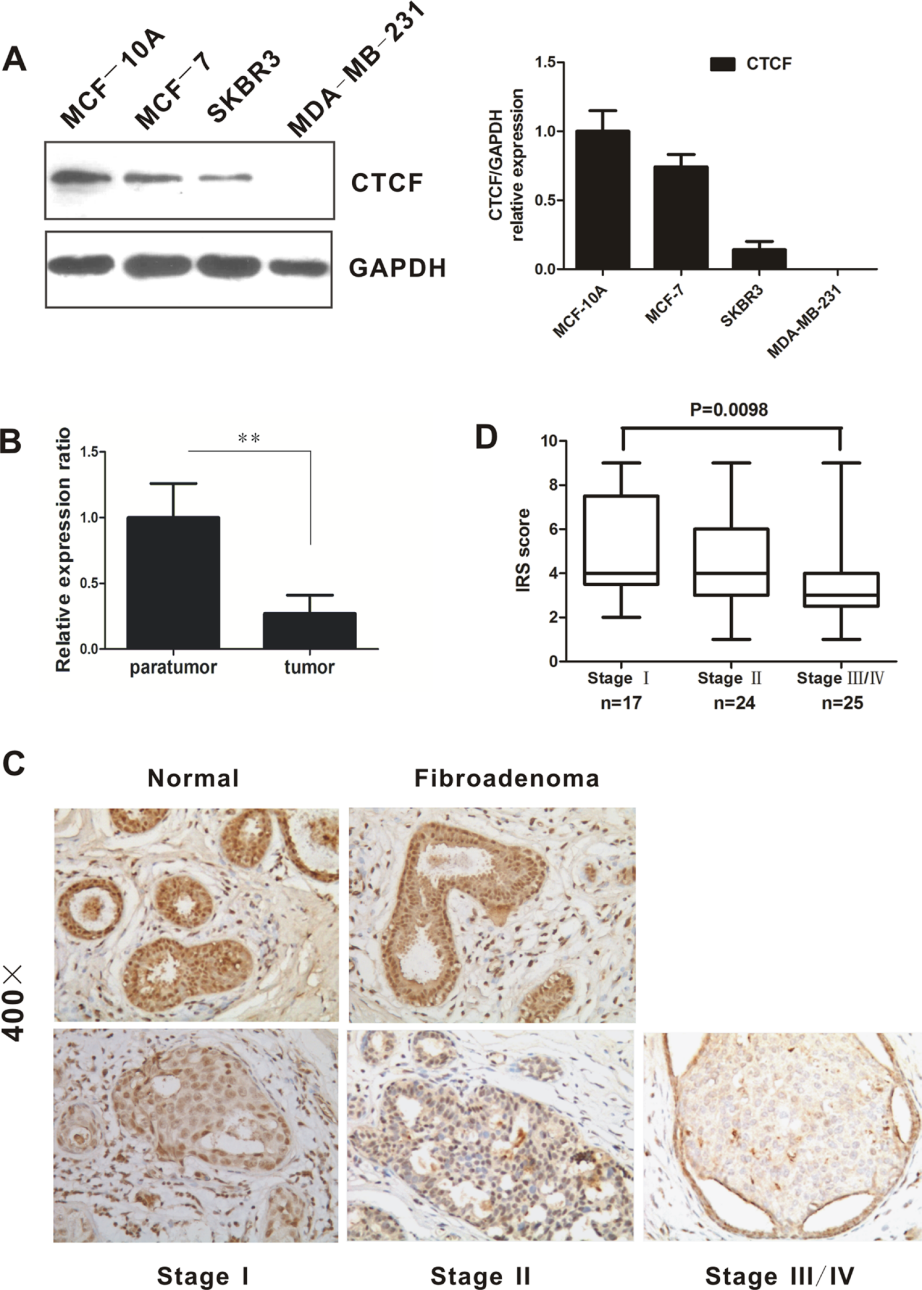
CTCF expression is downregulated in breast cancer tissues and cell lines (**A**) CTCF expression in human mammary epithelial cell line MCF-10A and human breast cancer cell lines (non-metastatic MCF-7 and metastatic SKBR3 as well as MDA-MB-231) was evaluated by western blotting analysis. GAPDH was used as an internal control. (**B**) Real-time quantitative PCR was used to detect the *CTCF* mRNA level in 20 fresh breast carcinoma tissues and corresponding peritumoral tissues. (**C**) Representative images of immunohistochemical staining for CTCF in normal breast tissues, fibroadenoma, and tumor tissues at the indicated clinical stages (×400). (**D**) Immunoreactive score (IRS) of CTCF protein expression in patients at different clinical stages. **indicates *P* < 0.01.

We first examined CTCF expression in human breast cancer cell lines MCF-7, SKBR3, and MDA-MB-231 as well as normal breast cells MCF-10A. Western blotting analysis showed that CTCF was downregulated in the three breast cancer cell lines examined (Figure 1A). However, no significant change in the *CTCF* mRNA level was observed in breast cancer cell lines as compared with that in MCF-10A (Supplementary Figure 1). The expression of CTCF was analyzed in only three breast cancer cell lines; therefore, further analysis was carried out to verify the difference in *CTCF* expression using normal and tumor breast tissues. Quantitative real-time reverse transcription PCR (qRT-PCR) indicated that the *CTCF* mRNA level was decreased significantly in human breast tumors (*n* = 20) compared with peritumoral tissues (Figure 1B). Moreover, immunohistochemical (IHC) analysis showed that the CTCF protein was downregulated significantly in breast cancer tissues (*n* = 66) compared with peritumoral tissues (*n* = 30) and fibroadenoma (*n* = 30) (Figure 1C, *P* < 0.01). These results suggested that CTCF expression is downregulated in breast cancer tissues and cell lines

### Correlation between CTCF expression and clinicopathological parameters in breast cancer

To investigate the clinical significance of CTCF downregulation in breast cancer, we analyzed the potential association between CTCF levels and clinical characteristics of paraffin-embedded breast cancer tissue specimens obtained from 66 patients (17, 24, and 25 cases at stage I, II, and III/IV, respectively) (Table 1). No significant correlation was observed between CTCF levels and age, estrogen receptor, progesterone receptor, or human epidermal growth factor receptor-2 (HER-2) status, whereas a low CTCF level was associated significantly with pTNM stage (*P* = 0.043) and pathological differentiation (*P* = 0.029). Furthermore, the immunoreactive score of Remmele and Stegner (IRS) statistics for CTCF also indicated that the level of CTCF was downregulated significantly in stage III/IV tumors compared with that in stage I tumors (Figure 1D, *P* = 0.0098).

**Table 1:** The association between CTCF expression and clinicopathological factors in patients with breast cancer

Characteristic	*N*	CTCF expression		*P* value
		High	Low	
Age				0.661
< 50	34	11	23	
> 50	32	12	20	
pTNM stage				0.043
I	17	8	9	
II	24	11	13	
III/IV	25	4	21	
Pathological differentiation				0.029
Well/moderate	43	19	24	
Poor	23	4	19	
ER status				0.147
Negative	31	8	23	
Positive	35	15	20	
PR status				0.450
Negative	30	9	21	
Positive	36	14	22	
HER-2 status				0.552
Negative	34	13	21	
Positive	32	10	22	

ER: estrogen receptor; PR: progesterone receptor; HER-2: human epidermal growth factor receptor-2.

To determine the effect of CTCF on survival of breast cancer patients, we generated Kaplan–Meier survival curve of 3,951 patients with breast cancer with low or high CTCF expression using Kaplan–Meier Plotter (www.kmplot.com/analysis). The results showed that higher *CTCF* mRNA expression in patients with breast cancer correlated with an improvement in relapse-free survival (Supplementary Figure 2).

### CTCF suppresses breast cancer progression

**Figure 2:**
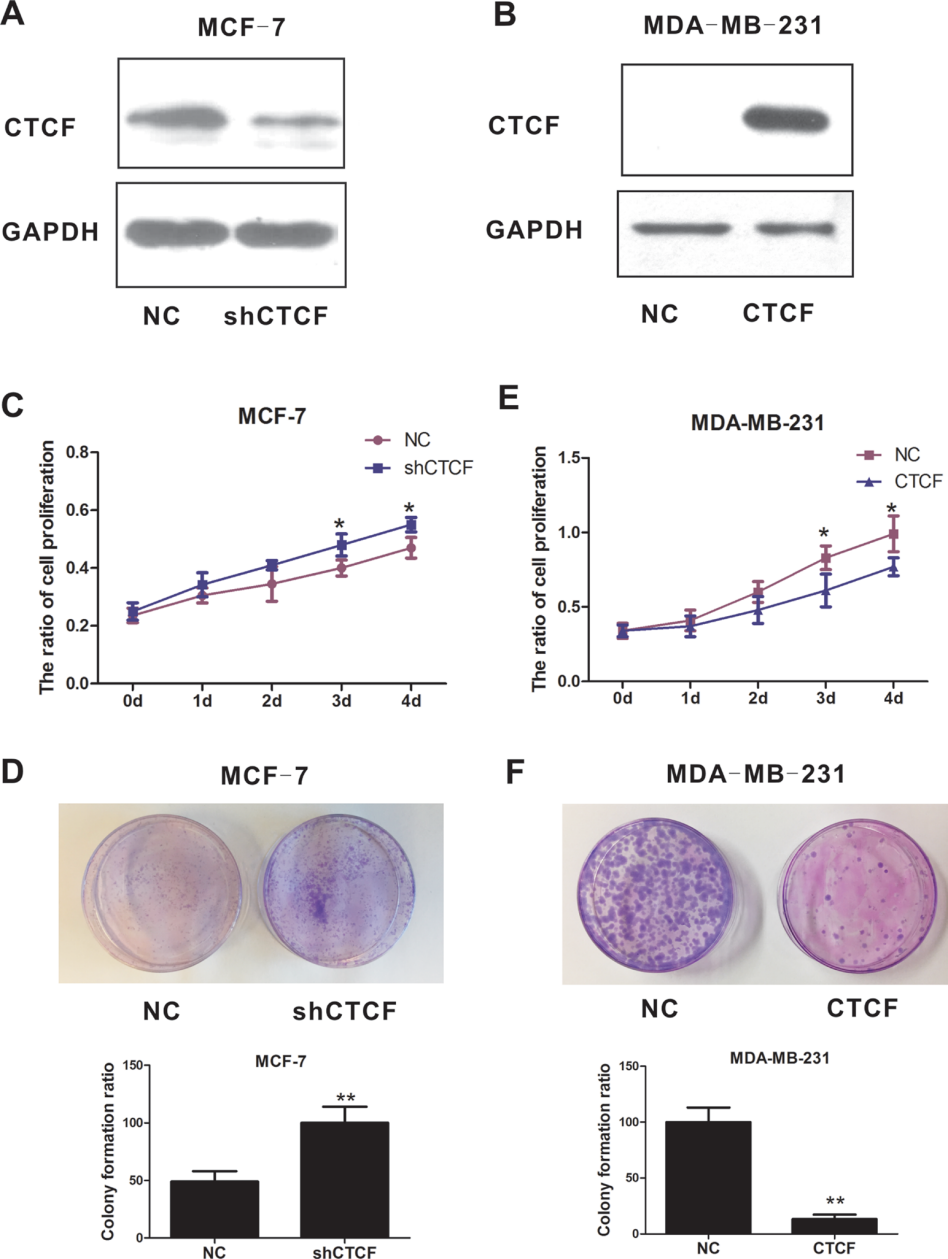
CTCF suppresses breast cancer cell proliferation and tumorigenicity Western blotting analysis confirmed *CTCF* knockdown in MCF-7 cells stably infected with lentiviral particles carrying the shCTCF vector (**A**) and overexpression in MDA-MB-231 cells stably infected with lentiviral particles carrying the *CTCF* vector (**B**). The effect of *CTCF* knockdown (**C**) and overexpression (**D**) on breast cancer cell proliferation was determined by the MTT assay. The effect of *CTCF* knockdown (**E**) and overexpression (**F**) on tumorigenicity of breast cancer cells was detected using a colony formation assay. *P indicates < 0.05, **indicates *P* < 0.01.

Our findings suggested an involvement of CTCF in breast cancer progression; therefore, we assessed the functional role of CTCF in the phenotype of breast cancer cells both *in vitro* and *in vivo*, via lentivirus-mediated knockdown and overexpression of *CTCF*, using MCF-7 and MDA-MB-231 cells lines as a model system. Given the significant difference in CTCF protein levels between non-metastatic MCF-7 and metastatic MDA-MB-231, we selected these two cell lines for subsequent functional studies. Knockdown of *CTCF* promoted MCF-7 tumorigenicity and cell proliferation (Figure 2A, 2C and 2D). By contrast, ectopic overexpression of *CTCF* attenuated MDA-MB-231 tumorigenicity and cell proliferation (Figure 2B, 2E and 2F).

**Figure 3:**
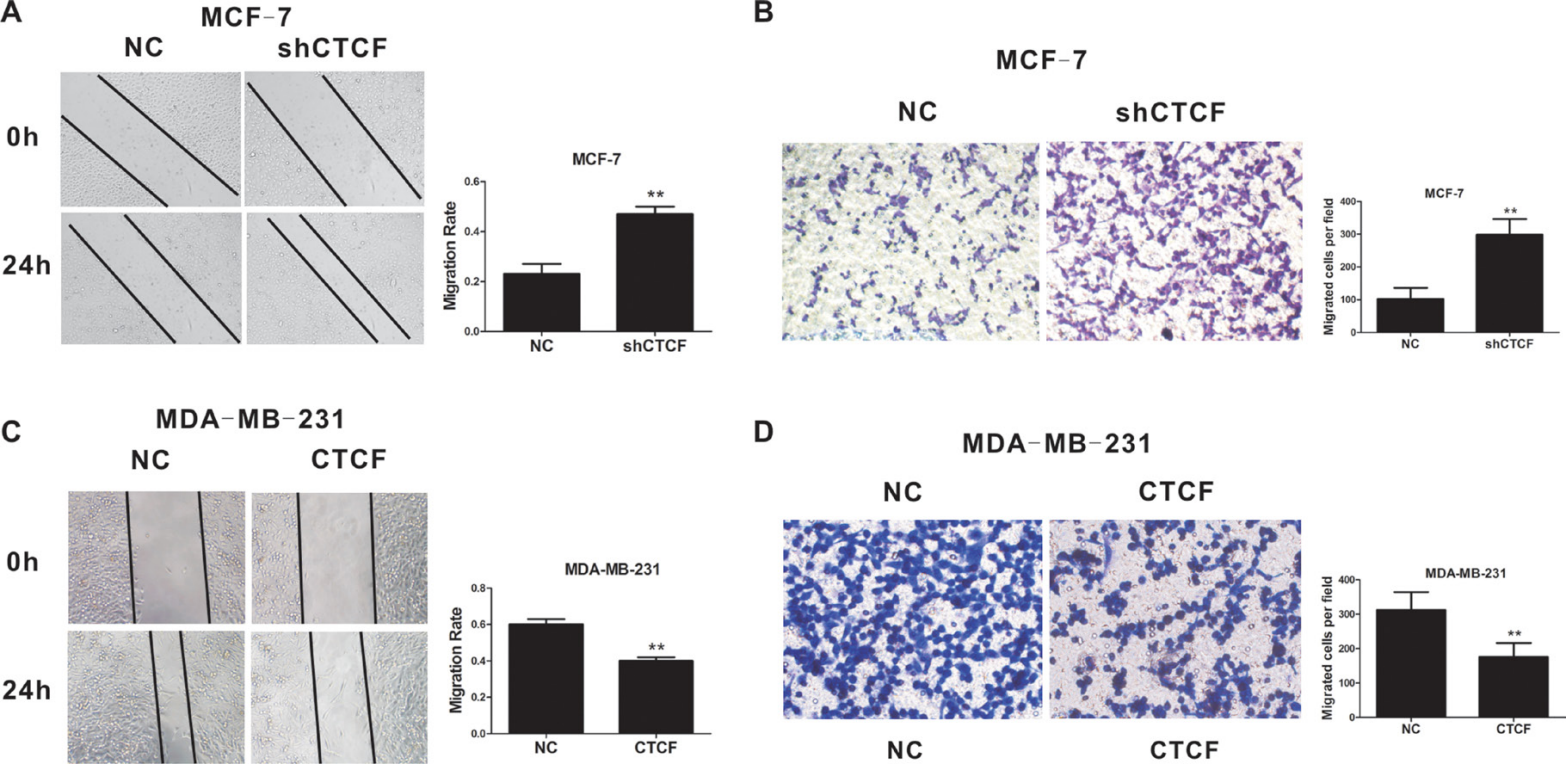
CTCF inhibits breast cancer cell migration and invasion Wound healing (**A** and **C**) and Transwell (**B** and **D**) assays were performed to detect the effect of *CTCF* knockdown (**A** and **B**) and overexpression (**C** and **D**) on migration and invasion in breast cancer cells. The left panel is a representative image and the right panel shows the average number per field. **indicates *P* < 0.01.

To determine if CTCF also inhibits metastasis and invasion in breast cancer, we performed wound healing and Transwell assays *in vitro*. As expected, *CTCF* knockdown enhanced MCF-7 cell migration and invasion (Figure 3A and 3B), while *CTCF* overexpression inhibited MDA-MB-231 cell migration and invasion (Figure 3C and 3D).

**Figure 4:**
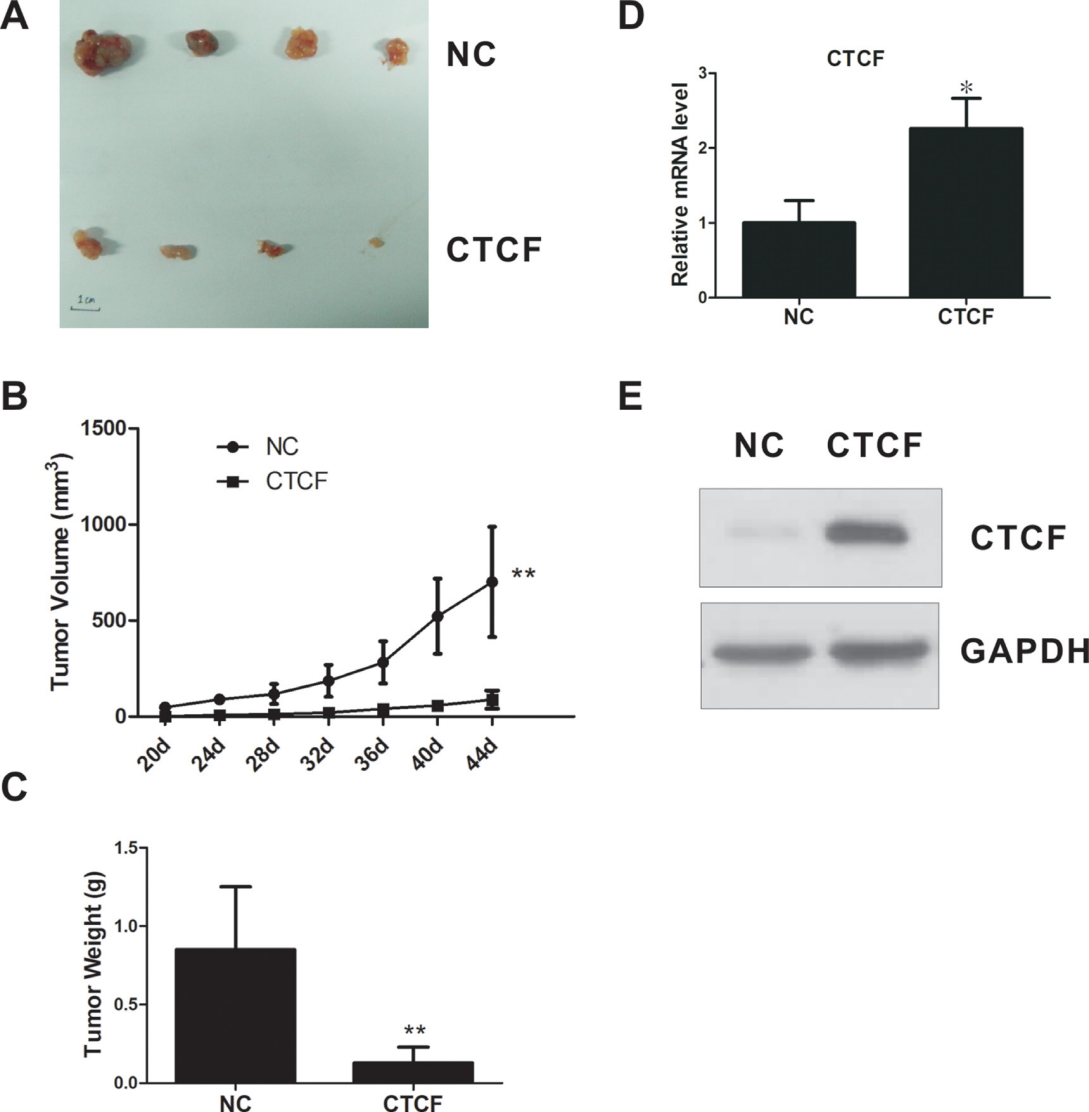
CTCF inhibits tumorigenicity of breast cancer cells *in vivo* (**A**) A xenograft nude mouse model was inoculated with either *CTCF*-overexpressing MDA-MB-231 cells or control cells (*n* = 5/group) and the tumor was isolated on day 44. (**B**) Xenograft tumor growth curves. (**C**) Mean weights of xenografts tumors. (**D** and **E**) CTCF mRNA and protein levels in xenograft tumors were measured using qRT-PCR and western blotting analysis. * indicates *P* < 0.05, **indicates *P* < 0.01.

To confirm the tumor suppressor role of CTCF *in vivo*, we established a xenograft model by inoculating nude mice with MDA-MB-231-CTCF or control cells. The *in vivo* assay indicated that enforced *CTCF* expression suppressed tumor growth significantly in the xenograft nude mouse model (Figure 4A–4E). Taken together, these results suggested that CTCF functions as a tumor suppressor and plays a pivotal role in breast cancer progression.

### CTCF downregulates multiple target genes of NF-κB by inhibiting the activation of RELA (p65)

**Figure 5:**
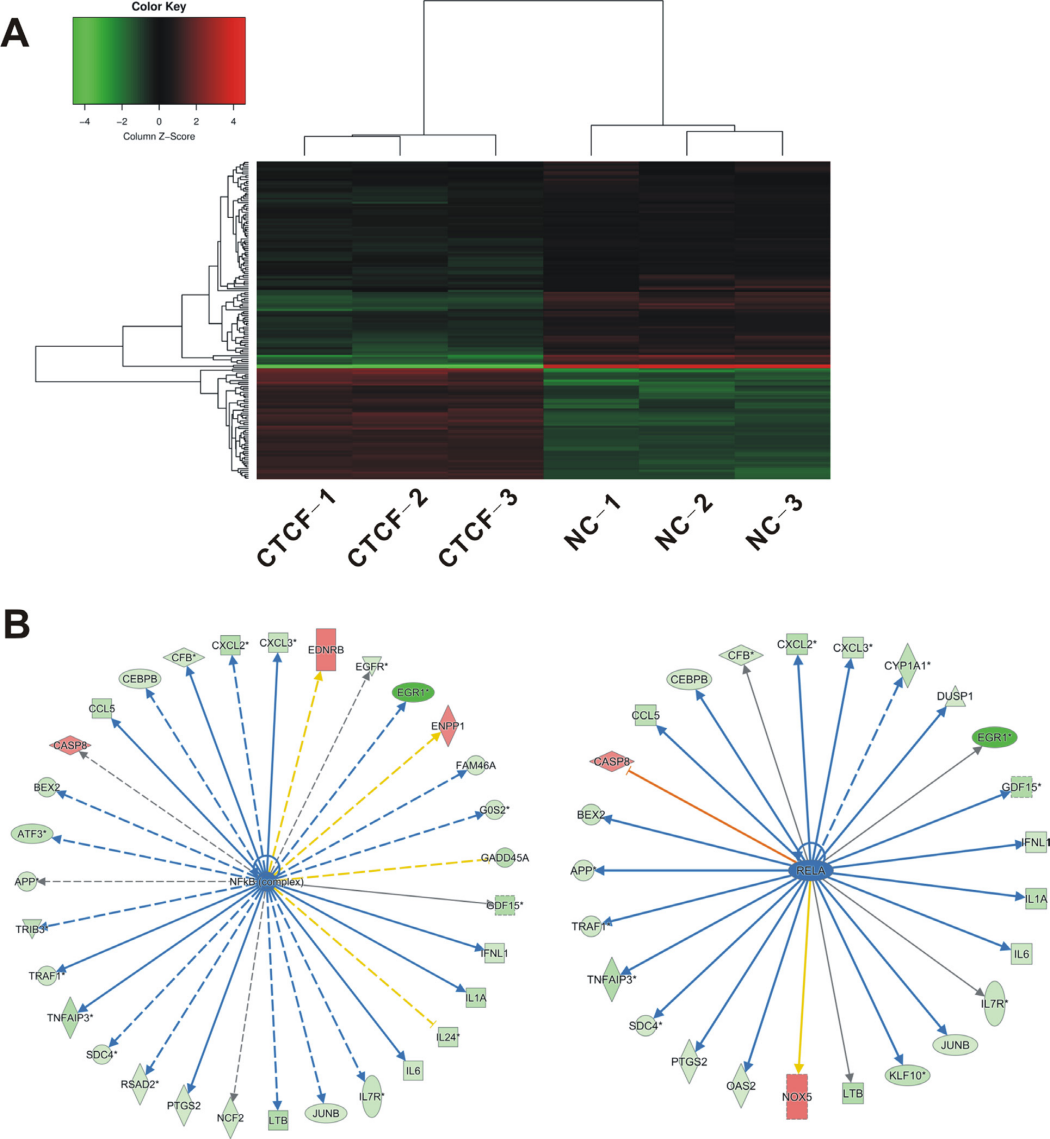
Identification of differentially expressed genes in MDA-MB-231 cells overexpressing CTCF Total RNA was extracted from the *CTCF*-overexpressing MDA-MB-231 cells and control cells, and gene expression microarray was performed as described in “Materials and Methods”. (**A**) Changes in gene expression > 1.3 fold and *P* < 0.05 are illustrated by a heat map. Green indicates relatively low expression and red indicates relatively high expression. (**B**) Of 198 differentially expressed genes, 36 are target genes of the NF-κB complex or RELA (p65). Green indicates downregulated genes and red indicates upregulated genes.

We next investigated the underlying mechanism involved in CTCF-mediated inhibition of migration and invasion in breast cancer cells. Using total RNA isolated from *CTCF*-overexpressing and control MDA-MB-231 cells, we performed a cDNA microarray analysis to identify differentially expressed genes and pathways induced by CTCF. The global gene expression profiling identified 198 differentially expressed genes (fold change > 1.3 and *P* < 0.05). Among them, 65 genes were upregulated and 133 genes were downregulated (Figure 5A, Supplementary Table 1). Biological functional analysis of these differentially expressed genes using gene ontology (GO) and pathway enrichment analyses identified the top 10 most suppressed pathways, including colorectal cancer metastasis signaling (Supplementary Figure 3). Furthermore, disease and functional analysis showed that the significantly activated functions included organismal death (Z-score = 3.409) and morbidity or mortality (Z-score = 3.383), while the significantly suppressed functions were migration of smooth muscle cells (Z-score = −3.373) and survival of the organism (Z-score = −3.332) (data not shown). Overall, these results suggested that CTCF might inhibit metastasis by regulating the expression of these genes.

**Figure 6:**
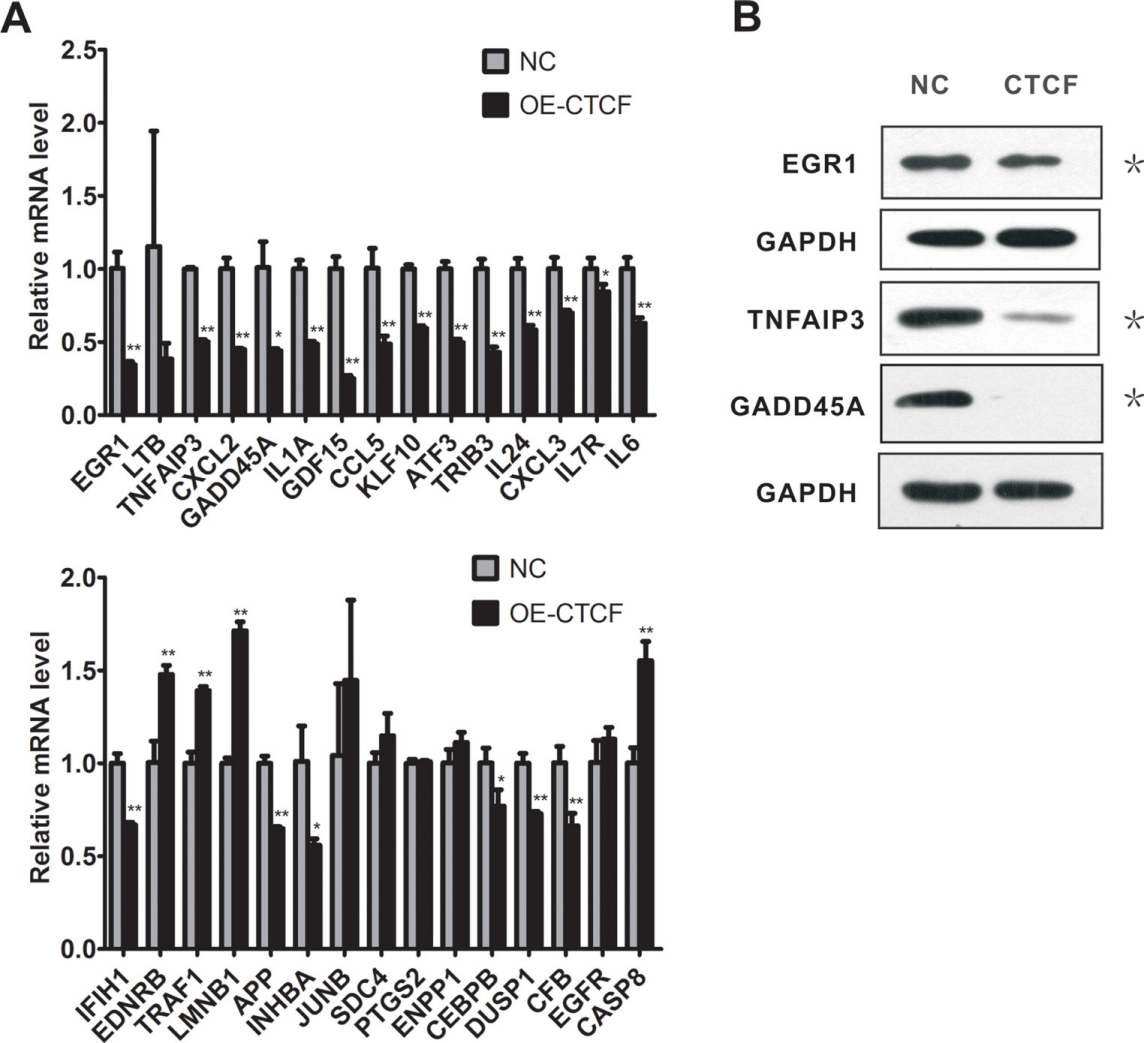
CTCF overexpression downregulates multiple NF-κB target genes in MDA-MB-231 cells (**A**) Real-time RT-PCR analysis of the expression of NF-κB target genes identified by the cDNA microarray in the *CTCF*-overexpressing and control MDA-MB-231 cells. (**B**) Western blotting analysis of the expression of three NF-κB target genes in the *CTCF*-overexpressing and control MDA-MB-231 cells. *indicates *P* < 0.05, **indicates *P* < 0.01.

We noted that more than 18% (36/198) of the differentially expressed genes corresponded to the target genes of NF-κB or RELA (p65) subunit, and most of these genes were downregulated upon *CTCF* overexpression (Figure 5B). In addition, analysis of the microarray data for upstream regulation factors revealed inhibition of genes involved in the activation of NF-κB signaling pathway, including NF-κB complex and the RELA (p65) subunit (Supplementary Figure 4). In the subsequent qRT-PCR validation, we selected 30 NF-κB target genes associated with proliferation and metastasis (Table 2). The qRT-PCR analysis showed that *CTCF* overexpression in MDA-MB-231 cells resulted in a significant downregulation of most of the NF-κB target genes (Figure 6A). Among the 30 target genes, the qRT-PCR results for 22 of them were consistent with the GeneChip results. We also detected the encoded protein levels of several selected genes, including early growth response protein 1 (EGR1), tumor necrosis factor alpha-induced protein 3 (TNFAIP3), and growth arrest and DNA-damage-inducible alpha (GADD45A), which are important NF-κB target genes and showed maximum changes in their expression (Table 2). Western blotting analysis showed that the protein levels of EGR1, TNFAIP3, and GADD45A were also downregulated in *CTCF*-overexpressing MDA-MB-231 cells (Figure 6B).

**Table 2:** Selected NF-κB target genes related to cell proliferation and metastasis influenced by CTCF overexpression in MDA-MB-231 cells

Gene symbol	Entrez gene ID	Fold change	*P* value
*EGR1*	NM_001964	−5.8429036	0.00072380295
*LTB*	NM_002341	−2.0426607	0.00080019515
*TNFAIP3*	NM_006290	−2.0378265	0.0002301113
*CXCL2*	NM_002089	−1.947091	0.00072380295
*GADD45A*	NM_001924	−1.9072746	0.00035669992
*IL1A*	NM_000575	−1.8493568	0.0006018482
*GDF15*	NM_004864	−1.8099316	0.0014306399
*CCL5*	NM_002985	−1.7817189	0.0014670709
*KLF10*	NM_005655	−1.7360209	0.0007831317
*ATF3*	NM_001030287	−1.7178577	0.0016058085
*TRIB3*	NM_021158	−1.7063142	0.0012432608
*IL24*	NM_001185156	−1.6383375	0.00093471195
*CXCL3*	NM_002090	−1.6109521	0.0018206392
*IL7R*	NM_002185	−1.5548722	0.00026126512
*IL6*	NM_000600	−1.4467697	0.0006270534
*IFIH1*	NM_022168	−1.4459864	0.000837096
*EDNRB*	NM_001201397	1.4439552	0.001778671
*TRAF1*	NM_005658	−1.3759115	0.0021421628
*LMNB1*	NM_005573	−1.357773	0.0010687009
*APP*	NM_201414	−1.3555276	0.005037326
*INHBA*	NM_002192	−1.3478045	0.005553174
*JUNB*	NM_002229	−1.3321716	0.013992447
*SDC4*	NM_002999	−1.332132	0.00073019817
*PTGS2*	NM_000963	−1.332128	0.0211235
*ENPP1*	NM_006208	1.3278843	0.016963318
*CEBPB*	NM_001285879	−1.3265668	0.009680476
*DUSP1*	NM_004417	−1.319924	0.0027198044
*CFB*	NM_001710	−1.3132958	0.00087543245
*EGFR*	NM_005228	−1.3061141	0.0036412638
*CASP8*	NM_001080125	1.3000749	0.0020934197

**Figure 7:**
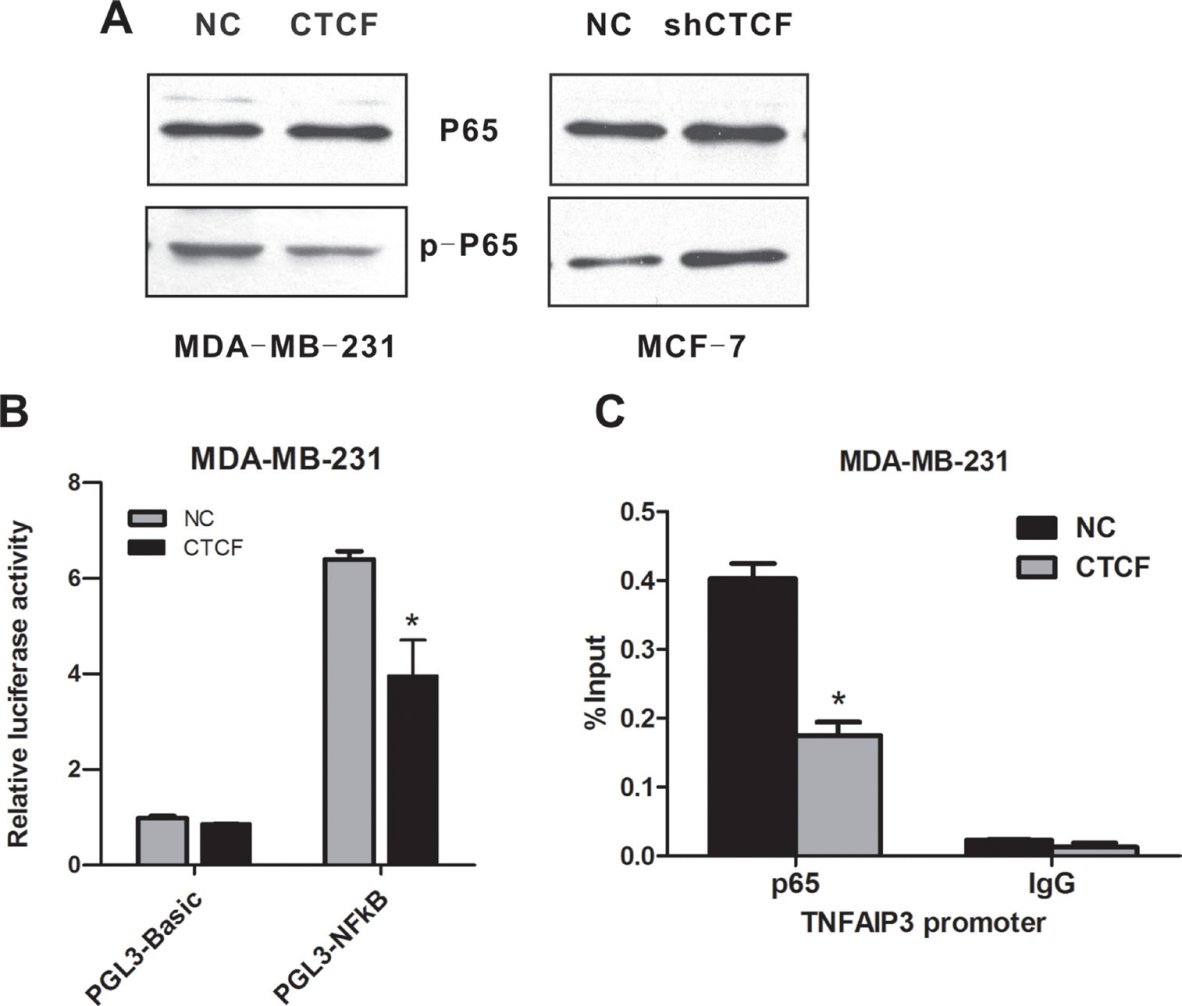
CTCF inhibits NF-κB (p65) activation (**A**) The levels of p65 and p-p65 in the *CTCF*-overexpressing MDA-MB-231 cells and *CTCF*-knockdown MCF-7 cells were detected using western blotting analysis. (**B**) *CTCF*-overexpressing and control MDA-MB-231 cells were transiently transfected with either empty vector pGL3-Basic or luciferase reporter pGL3-NFkB bearing six canonical NF-kB binding sites. Forty-eight hours after transfection, the luciferase activity was measured. (**C**) ChIP analysis was performed to detect p65 occupancy at the *TNFAIP3* promoter in the *CTCF*-overexpressing and control MDA-MB-231 cells. Data represent three independent experiments. Error bars represent the standard error of the mean. *indicates *P* < 0.05.

As NF-κB p65 subunit phosphorylation is the main marker of NF-κB activation, we examined the effect of CTCF on p65 phosphorylation in MCF-7 and MDA-MB-231 cells. Western blotting analysis showed that the ectopic overexpression of *CTCF* attenuated p65 phosphorylation, whereas knockdown of *CTCF* promoted p65 phosphorylation (Figure 7A). Moreover, the luciferase assay using a luciferase reporter construct bearing six canonical NF-κB p65 binding sites indicated that the downregulation of phospho-p65 (Ser536) induced by *CTCF* overexpression resulted in a significant reduction in luciferase activity (Figure 7B). To further establish the physical and functional interaction between p65 Ser536 and the differentially expressed NF-κB target genes, we performed a chromatin immunoprecipitation (ChIP) assay using the *TNFAIP3* gene as an example. Real-time ChIP-PCR showed that the occupancy of the *TNFAIP3* promoter by p65 was decreased in MDA-MB-231 cells in response to *CTCF* overexpression (Figure 7C).

## DISCUSSION

Several studies have identified that *CTCF* deletions or mutations are associated with human breast carcinogenesis [23, 25]. However, the precise role of CTCF in breast cancer and the underlying molecular mechanism remain largely unknown. In this study, we showed that CTCF protein levels, but not mRNA expression, were downregulated in breast cancer cells, indicating that post-transcriptional CTCF regulation plays an important role in breast cancer cells. In addition, CTCF levels in breast cancer lesions were significantly lower than those in peritumoral tissues. IHC analysis showed that the decrease in CTCF level was associated significantly with pTNM stage and pathological differentiation. Analysis of the publicly available datasets (Kaplan-Meier Plotter) revealed a correlation between a decreased CTCF mRNA level and poor prognosis in breast cancer patients. However, we found no significant difference in CTCF mRNA levels in three breast cancer cell lines examined. The differences between cell lines and tissues may be explained by the differences in cellular environment, cell type and genetic background. Taken together, these findings provided strong evidence that the downregulation of CTCF enhances breast cancer progression.

In agreement with previous studies in other human cancers [24, 26], we found that CTCF might inhibit cell proliferation in breast cancer cells. We reported, for the first time, that *CTCF* knockdown increased cell migration and invasion in breast cancer cells. By contrast, CTCF overexpression markedly reduced the migration and invasion of cancer cells *in vitro* and tumorigenicity *in vivo*. Thus, our *in vivo* results were consistent with those observed *in vitro*, further confirming that CTCF suppresses the tumorigenicity of breast cancer cells. Our findings indicated a tumor suppressor role of CTCF in the development and progression of breast cancer. A recent study reported that CTCF exhibits oncogenic activity that enhances the aggressiveness and progression of neuroblastoma by promoting growth, invasion, and metastasis in neuroblastoma cells [27]. Thus, CTCF may play different roles in different cancers.

Aberrant or constitutive activation of NF-kB in breast cancer cells is associated with high malignancy and the promotion of osteolytic bone metastasis [28, 29]. RELA (p65), the major transactivating subunit of NF-κB, is responsible for the activation of downstream genes that participate in multiple cellular processes related to carcinogenesis [30]. Transcriptional profile analysis and real-time PCR verification revealed the downregulation of several NF-κB target genes in *CTCF*-overexpressing MDA-MB-231 cells. Microarray data analysis of upstream regulation factors indicated the CTCF-mediated inhibition of the NF-κB complex and RELA (p65) activation, which was validated by the luciferase reporter assay. CTCF is thought to decrease p65 phosphorylation and attenuate its DNA-binding ability. The mechanisms underlying CTCF’s inhibition of NF-κB activity will be explored in future studies, including the possibility of physical interaction. Does CTCF bind to NF-kB promoter/enhancer region? Does it need co-activators/co-repressors? These questions are all the directions of our future studies. The results of the present study suggested that the CTCF/NF-κB pathway might be involved in the development of breast cancer. Interestingly, *CTCF* has been reported as a downstream target gene of the NF-κB pathway and NF-κB has been shown to mediate the upregulation of *CTCF* expression directly [31, 32]. Thus, our results suggest that it would be reasonable to investigate whether CTCF and NF-κB exist in a feedback regulatory loop and to analyze their regulatory effect on each other.

In this study, CTCF induced the inhibition of several NF-κB target genes, which further confirmed the CTCF-mediated inhibition of NF-κB activity. The majority of the altered target genes are associated with proliferation and metastasis. The most affected genes included *TNFAIP3*, *EGR1*, and *GADD45a*. *TNFAIP3*, also known as A20, encodes an ubiquitin-editing enzyme and its overexpression is associated with breast cancer aggressiveness [33]. EGR1 is a mammalian nuclear transcription factor with an indispensable role in breast cancer proliferation, migration, chemoinvasion, and angiogenesis [34–36]. GADD45a is reported to promote Myc-driven breast cancer, resulting in increased tumor vascularization and growth [37]. Taken together, CTCF acts as a tumor suppressor in breast cancer by inhibiting the activation of NF-κB (p65) and its target genes. Thus, CTCF might be an effective therapeutic target for the treatment of breast cancer.

## MATERIALS AND METHODS

### Cell culture and plasmid constructs

MCF-7, MDA-MB-231, and 293T cells were maintained in Dulbecco’s Modified Eagle’s Medium (DMEM, GIBCO). SKBR3 cells were cultured in Roswell Park Memorial Institute (RPMI)-1640 medium (GIBCO). All media were supplemented with 10% fetal bovine serum (FBS, HyClone). The human epithelial breast cell line, MCF-10A, was maintained in DMEM containing 0.5 μg/mL hydrocortisone, 10 μg/mL insulin, 20 ng/mL human epidermal growth factor (EGF), and 5% heat-inactivated horse serum. All cell lines were obtained from the Cell Resource Center, Peking Union Medical College. Cell lines were free of mycoplasma contamination, according to PCR analysis. The species of origin was confirmed using PCR, and the identity of the cell lines was authenticated using short tandem repeat (STR) profiling (FBI, CODIS). The full-length *CTCF* coding sequence was synthesized chemically and subcloned into lentivirus vector GV358 (GeneChem, Shanghai, China). MDA-MB-231 cells stably expressing *CTCF* and control cells were established by lentivirus-mediated gene transfer.

### Tissue samples

We analyzed retrospectively the clinicopathological data of 66 patients diagnosed with breast cancer who underwent surgical resection at the Department of Breast surgery, Peking Union Medical College Hospital from January to October 2014. All specimens were determined as invasion ductal carcinoma by pathological examinations. Informed consent was obtained from each patient included in this study, and the study, along with the procedures used, was approved by the Institutional Research Ethics Committee of PUMCH. Tumors were classified histologically according to the TNM staging criteria from the American Joint Committee on Cancer (AJCC). Tumor grade was evaluated as per the World Health Organization (WHO) criteria.

### Immunohistochemistry (IHC) assay

Immunohistochemistry was performed according to the standard protocol. Briefly, sections of paraffin-embedded tissue (4-μm thick) were deparaffinized with xylene and hydrated in gradient diluted alcohol solutions. Endogenous peroxidase was blocked by incubation in 3% hydrogen peroxide. High-pressure cooking was applied to retrieve the antigen and 5% normal goat serum was used to block non-specific protein binding. Slides were incubated with the primary anti-CTCF antibodies (1:100, Millipore) overnight at 4°C, followed by probing with anti-rabbit secondary antibodies for 1 h. Finally, all slides were stained with diaminobenzidine and counterstained with hematoxylin. Slides incubated with phosphate-buffered saline (PBS) instead of primary antibody were used as the negative control. The intensity of staining was graded according to the following criteria: 0 (no staining), 1 (weak staining = light yellow), 2 (moderate staining = yellow brown), and 3 (strong staining = brown). The proportion of positive-staining tumor cells was scored as follows: 0 (no positive cells), 1 (< 10% positive cells), 2 (10–50% positive cells), and 3 (> 50% positive cells). The IRS was calculated by multiplying the staining intensity and staining-positive proportion scores, with an IRS ≤ 4 considered as low expression and > 4 as high expression.

### RNA isolation and quantitative reverse transcription polymerase chain reaction (qRT-PCR)

Total RNA was extracted using the Trizol reagent (Life Technologies, Carlsbad, CA, USA) and first strand complementary DNA (cDNA) generated by the Reverse Transcription System (Takara) in a 20-µL reaction containing 2 µg total RNA, according to the manufacturer’s protocol. Real-time PCR was performed on a Roche LightCycler 480 using a SYBR Green reaction mix (Takara). Values are shown means and standard deviations (SD) of the results from at least three independent experiments. All primer sets are available upon request.

### RNA interference and lentivirus package

Cell lines stably expressing CTCF and negative control (NC) small hairpin RNA (shRNA) were established using a lentivector-based shRNA expression system. CTCF- or NC-shRNA were inserted into the lentivirus expression plasmid GV248 (GeneChem, Shanghai, China) with the following sequences: shCTCF, 5′-GGTGTAAAGAAGACATTCC-3′ and non-silencing shRNA, 5′-TTCTCCGAACGTGTCACGT-3′. Plasmids were transfected into 293T cells along with packaging plasmids pHelper 1.0 and pHelper 2.0 (pVSVG-I and pCMVΔR 8.92 plasmids, respectively). Viral supernatants were collected after 48 h of transfection to infect breast cancer cells MCF-7, as described in the supplier’s protocol. *CTCF* knockdown efficiency was determined by qRT-PCR and western blotting.

### Western blotting analysis

Whole cell lysates were used for western blotting analysis as previously described [38, 39], with glyceraldehyde 3-phosphate dehydrogenase (GAPDH) used as a loading control. The anti-CTCF antibody (07–729) was obtained from Millipore, while antibodies against EGR1 (ab54966), TNFAIP3 (ab74037), GADD45A (ab180768), and p65 (ab16502) were obtained from Abcam; a GAPDH monoclonal antibody (SC-32233) and rabbit or mouse secondary antibodies were purchased from Santa Cruz Biotechnology. The anti-phospho-NF-κB p65 (Ser536) (93H1) antibody was obtained from Cell Signaling.

### 3-(4,5-dimethylthiazol-2-yl)-2,5-diphenyltetrazolium bromide (MTT) assay

MCF-7 or MDA-MB-231 cells were seeded at 5,000 cells/well into 96-well plates in 200 μL fresh medium. After incubation for an appropriate interval, a 20-μL stock MTT solution (5 mg/mL) was added to each well. Following incubation for 4 h, the medium was aspirated and cells were treated with 150 μL dimethyl sulfoxide for 10 min. The absorbance of each well was measured at a wavelength of 490 nm. All experiments were conducted in triplicate and repeated three times.

### Colony formation assays

MCF-7 or MDA-MB-231 cells were plated at 500 or 100 cells into 60-mm dishes and cultured for 7 or 14 days, respectively. Colonies were fixed with methyl alcohol for 30 min, stained with Giemsa for 30 min, and counted. Colonies containing at least 50 cells were scored. Each assay was performed in triplicate.

### Transwell assay

Cell invasion was assessed using the Transwell assay. MCF-7 or MDA-MB-231 cells were harvested after trypsinization and washed with a serum-free medium containing 0.1% bovine serum albumin. Cells were diluted to 5 × 10^5^ cells/mL and 100 μL of the cell suspension was seeded on the top of Matrigel invasion chambers (8 μm pore size, Corning, Cat No. 354480). The lower chamber was filled with 600 μL medium containing 10% FBS. After incubation for 24 h at 37°C, cells from the upper chamber were gently removed with a cotton swab. The filter was fixed with 4% paraformaldehyde for 15 min, stained with 500 μL 0.1% crystal violet for 15 min, washed with PBS, and counted under a light microscope to determine cell invasion. All experiments were conducted in triplicate and repeated three times.

### Wound healing assay

MCF-7 or MDA-MB-231 cells were seeded into a 6-well plate and grown to 90% confluence in a complete medium. Cells were wounded using a plastic tip, washed with PBS to remove cell debris, and incubated in low serum medium (0.5% FBS) for up to 24 h. Cells were photographed under a microscope equipped with a camera at different time points.

### Xenograft tumor model

Female BALB/c-nude mice (4–5 weeks old, 14–16 g) were purchased from the Charles River Laboratories of Beijing and housed in barrier facilities with a 12 h/12 h light/dark cycle. All mouse experiments were performed according to the institutional guidelines and approved by the Institutional Animal Care and Use Committee (IACUC). Mice were divided randomly into two groups (*n* = 5/group). For tumor cell implantation, 5 × 10^6^ MDA-MB-231 cells (100 μL volume) transfected with CTCF or NC lentivirus were injected subcutaneously into the mice. Tumors were detected on day 20 after injection and then examined once every 4 days. Tumor length, width, and thickness were measured using a caliper and tumor volume was calculated by the ellipsoid volume calculation formula: 0.5 × (length × width^2^). At day 44 after injection, animals were euthanized and the tumors excised and weighed.

### GeneChip microarray analysis and data normalization

Total RNA from MDA-MB-231 cells transfected with NC- or CTCF-OE-lentivirus (*n* = 3) was extracted using the Trizol reagent. A NanoDrop 2000 and Agilent Bioanalyzer 2100 were used for quantitative and qualitative RNA detection, respectively. The GeneChip PrimeView Human Gene Expression Array (Affymetrix) was used for microarray processing to determine the gene expression profile, according to the manufacturer’s instruction. Significant differences in gene expression between MDA-MB-231 cell treated with NC- and CTCF-OE-lentivirus were identified as follows: *P* < 0.05 and the absolute fold change > 1.3. The GeneChip data has been submitted to the NCBI Gene Expression Omnibus and is accessible through GEO series accession number GSE98337.

### Luciferase reporter assays

Analysis of NF-kB activity was performed by cloning six canonical NF-kB RELA (p65) binding sites upstream of the luciferase gene in the pGL3-basic reporter vector. MDA-MB-231 cells overexpressing *CTCF* were transfected with the luciferase reporter and pRL-TK plasmid. After 48 h, cells were lysed with passive lysis buffer and the lysates were analyzed for both firefly and *Renilla* luciferase activity using a dual-luciferase reporter assay kit (Promega). Luciferase activity was normalized for transfection efficiency using the pRL-TK reporter (Promega) as an internal control. A Centro XS³ LB 960 Microplate Luminometer (Berthold) was used according to the manufacturer’s instructions. The results are expressed as the percentages of relative luciferase activities of the control, which was set as 100%.

### Chromatin immunoprecipitation (ChIP)

The chromatin IP assay was performed as described previously [38]. For quantification of the ChIP assay, real-time PCR was performed with SYBR Green dye on an iCycler iQ (Bio-Rad) system. All quantitative PCR signals from the IP samples were normalized to that of the respective input samples. Values shown are means and SD of the results from ≥ 3 independent experiments. The anti-p65 antibody (ab7970) for ChIP was obtained from Millipore. Primers used for the *TNFAIP3* promoter were 5′-CAGCCCGACCCAGAGAGTCAC-3′ and 5′-CGGGCTCCAAGCTCGCTT-3′.

### Statistical analysis

Data are presented as mean ± SD of at least three independent experiments for each group. A chi-squared exact test and Spearman’s correlation were applied to analyze the association between CTCF expression and various clinicopathological parameters. Differences were compared using two-sample *t*-tests or analysis of variance (ANOVA) for independent samples. For all analyses, a value of *P* < 0.05 was defined as statistically significant.

## SUPPLEMENTARY MATERIALS FIGURES AND TABLE




