# Supplementary material for: CCCTC-binding factor inhibits breast cancer cell proliferation and metastasis via inactivation of the nuclear factor-kappaB pathway
Source: Oncotarget. 2017 Jul 4;8(55):93516–29. doi: 10.18632/oncotarget.18977 (PMC5706815; doi:10.18632/oncotarget.18977)
Supplement: Supplementary file 1 [file oncotarget-08-93516-s001.pdf]

# CCCTC-binding factor inhibits breast cancer cell proliferation and metastasis via inactivation of the nuclear factor-kappaB pathway

## SUPPLEMENTARY MATERIALS

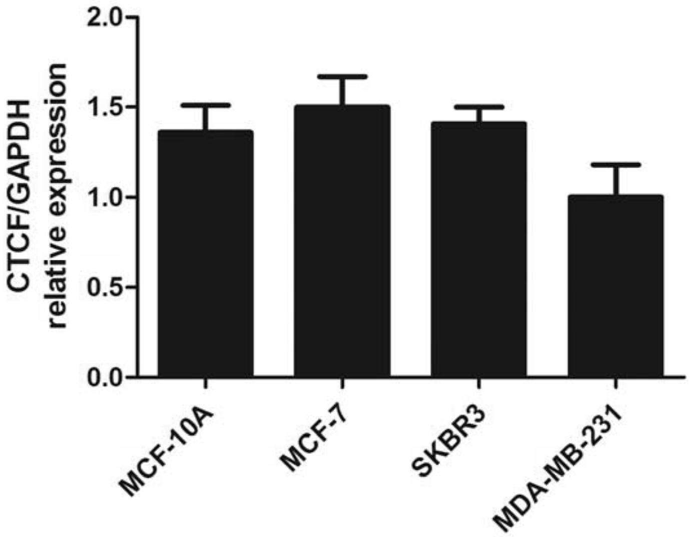


**Supplementary Figure 1: CTCF mRNA expression in breast cancer cells.** qRT- PCR was applied to detect CTCF mRNA expression in breast cancer cells and normal breast epithelial MCF-10A cells. The data are represented as mean ± standard deviation (SD) from three independent experiments.

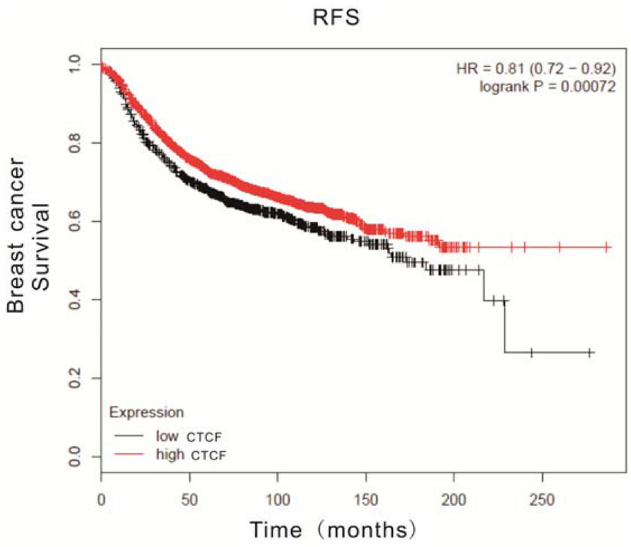


**Supplementary Figure 2: Prognostic significance of CTCF in breast cancer.** The effect of CTCF mRNA expression level on the relapse-free survival (RFS) in 3951 breast cancer patients was analyzed and the Kaplan-Meier survival curve were generated by Kaplan-Meier Plotter (http://www.kmplot.com).

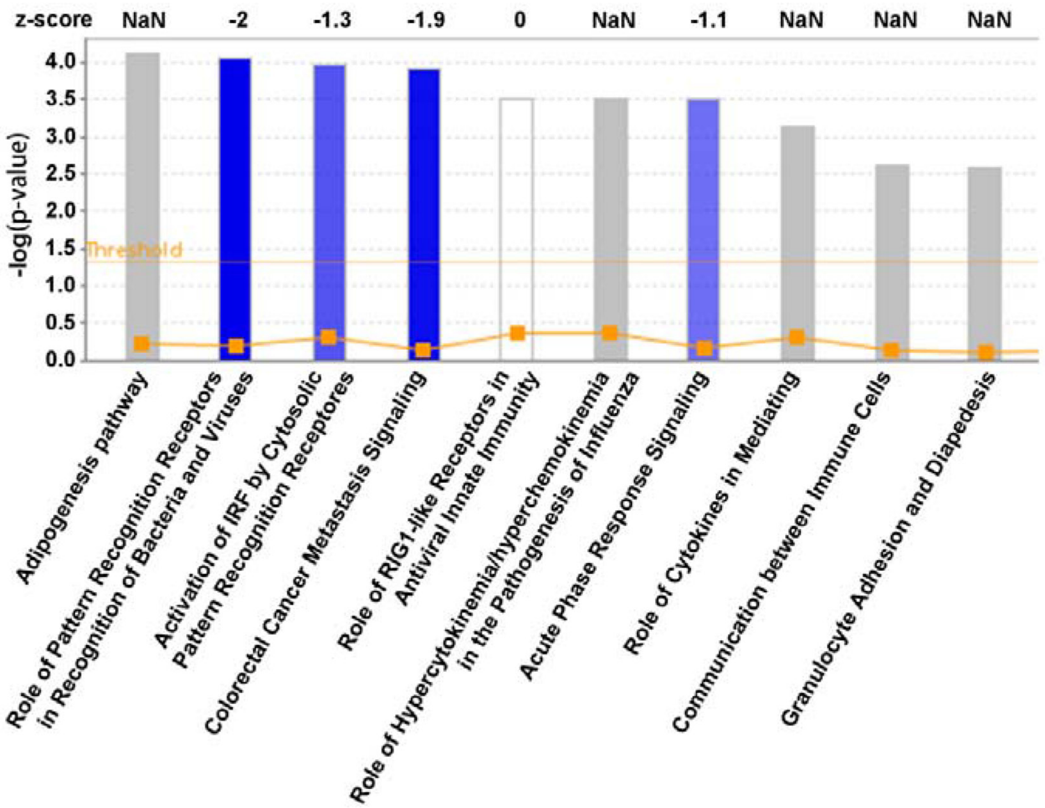


**Supplementary Figure 3: Enrichment of differential gene in classical signal pathway.** The x-axis shows pathways category and the y-axis shows -Log (*P*-value).The larger -Log (*P*-value) indicated a smaller *P* value.

| Upstream Regulator | Exp Fold Change | Molecule Type | Predicted Activation State | Activation z-score | p-value of overlap | Target molecules in dataset | Mech Net |
|---|---|---|---|---|---|---|---|
| lipopolysaccharide | | chemical drug | Inhibited | -5.5 | 7.85E-30 | ANGPTL2,APOL6,APP,ARG2,A | 87 |
| IFNG | | cytokine | Inhibited | -5.094 | 1.36E-23 | APOL6,APP,ARG2,ASNS,ATF3 | 79 |
| TLR3 | | transmembrane receptor | Inhibited | -3.78 | 1.82E-23 | ARG2,ARRDC4,ATF3,CCL5,CF | 61 |
| poly rI:rC-RNA | | biologic drug | Inhibited | -5.137 | 1.17E-21 | ATF3,CCL5,CEBPB,CFB,CXCL | 73 |
| TICAM1 | | other | Inhibited | -3.74 | 1.59E-19 | CCL5,CFB,CH25H,CXCL2,CXC | 58 |
| Salmonella enterica serotype | | chemical toxicant | Inhibited | -3.588 | 4.84E-19 | ARRDC4,ASNS,CCL5,CFB,CX | 84 |
| TNF | | cytokine | Inhibited | -4.937 | 8.66E-19 | APP,ATF3,BTG1,CASP8,CCL5, | 84 |
| NFkB (complex) | | complex | Inhibited | -3.714 | 1.94E-18 | APP,ATF3,BEX2,CASP8,CCL5, | 80 |
| RELA | | transcription regulator | Inhibited | -4.055 | 1.44E-17 | APP,BEX2,CASP8,CCL5,CEBP | 81 |
| STAT3 | | transcription regulator | | -0.217 | 3.55E-17 | ARG2,BEX2,CCL5,CEBPB,CFB | 78 |
| U0126 | | chemical - kinase inhibitor | Activated | 2.475 | 5.39E-17 | ARG2,ATF3,CA9,CCL5,CEBPB, | 79 |
| beta-estradiol | | chemical - endogenous mammalian | | -1.345 | 6.6E-17 | ABLIM1,APP,ATF3,BEX2,BTG1 | 88 |
| TGFB1 | | growth factor | Inhibited | -3.11 | 9.92E-17 | APP,ASNS,BTG1,CASP8,CCL5 | 96 |
| STAT1 | | transcription regulator | Inhibited | -2.468 | 1.47E-16 | APOL6,BTG1,CASP8,CCL5,CF | 71 |
| IFNLR1 | | transmembrane receptor | | | 1.54E-16 | ATF3,CCL5,CXCL2,CXCL3,IFIH | 59 |
| MAPK1 | | kinase | | 0.739 | 1.75E-16 | APOL6,ARG2,ATF3,CCL5,CFB, | 74 |
| IFNB1 | | cytokine | Inhibited | -2.638 | 2.38E-16 | CASP8,CCL5,CH25H,CXCL2,C | 63 |

**Supplementary Figure 4: The top regulators (Ranked by *P*-value) in IPA analysis of expression profiles in CTCF over-expressing and control MDA-MB-231 cells.** Transcriptional profiling in CTCF over-expressing and control MDA-MB-231 cells was carried out by hybridizing to Affymetrix GeneChip PrimeView Human Gene Expression Array (901838) and the chip data were analyzed with the Ingenuity Pathway Analysis Software (IPA). NF-κB pathway including NF-κB complex and RELA (p65) subunit were affected by the over-expression of CTCF.

**Supplementary Table 1: 198 differentially expressed genes were identified by the gene expression profiling analysis.** See Supplementary_Table_1
